# Integration of AI and ML in Tuberculosis (TB) Management: From Diagnosis to Drug Discovery

**DOI:** 10.3390/diseases13060184

**Published:** 2025-06-11

**Authors:** Sameeullah Memon, Shabana Bibi, Guozhong He

**Affiliations:** 1 Institute of Health, Kunming Medical University, Kunming 650500, China; heguozhong@kmmu.edu.cn; 2 Department of Biosciences, Shifa Tameer-e-Millat University, Islamabad 44000, Pakistan; shabana.bibi.stmu@gmail.com

**Keywords:** tuberculosis, artificial intelligence, sputum smear microscopy, culture tests, chest X-rays, deep learning, machine learning

## Abstract

Tuberculosis (TB) is an infectious disease caused by Mycobacterium tuberculosis. Despite the improvements in diagnostic techniques, the accuracy of TB diagnosis is still low. In recent years, the development of artificial intelligence (AI) has opened up new possibilities in diagnosing and treating TB with high accuracy compared to traditional methods. Traditional diagnostic techniques, such as sputum smear microscopy, culture tests, and chest X-rays, are time-consuming, with less sensitivity for the detection of TB in patients. Due to the new developments in AI, advanced diagnostic and treatment techniques have been developed with high accessibility, speed, and accuracy. AI, including various specific methodologies, is becoming vital in managing TB. Machine learning (ML) methodologies, such as support vector machines (SVMs) and random forests (RF), alongside deep learning (DL) technologies, particularly convolutional neural networks (CNNs) for image analysis, are employed to analyze diverse patient data, including medical images and biomarkers, to enhance the accuracy and speed of tuberculosis diagnosis. This study summarized the benefits and drawbacks of both traditional and AI-driven TB diagnosis, highlighting how AI can support traditional techniques to increase early detection, lower misdiagnosis, and strengthen international TB control initiatives.

## 1. Introduction

### 1.1. Overview of TB

Tuberculosis (TB) is an infectious disease referred to as the “white death” typically caused by the Mycobacterium tuberculosis (MTB) bacteria [1]. TB primarily impacts the lungs, though it can also affect other body parts. The phrase “latent TB” refers to diseases that usually do not show symptoms [2]. Roughly 10% of dormant illnesses become acute diseases because of the lack of treatment, and kill about half of those affected [3]. Common symptoms of active TB include fever, night sweats, weight loss, and a chronic cough with bloody mucus. The infection of various organs can result in a variety of symptoms. Individuals with active lung TB can spread the illness to others by sneezing, coughing, spitting, or speaking [4]. Individuals with HIV/AIDS and smokers are more likely to be infected. Traditional methods, such as chest X-rays, microscopic analysis, and culture of body fluids, are used to detect TB. Blood tests and the tuberculin skin test (TST) are used to diagnose latent TB [5]. TB prevention includes screening for high-risk persons, early identification and treatment of cases, and the bacillus Calmette–Guérin (BCG) vaccination [6].

**Figure 1:**
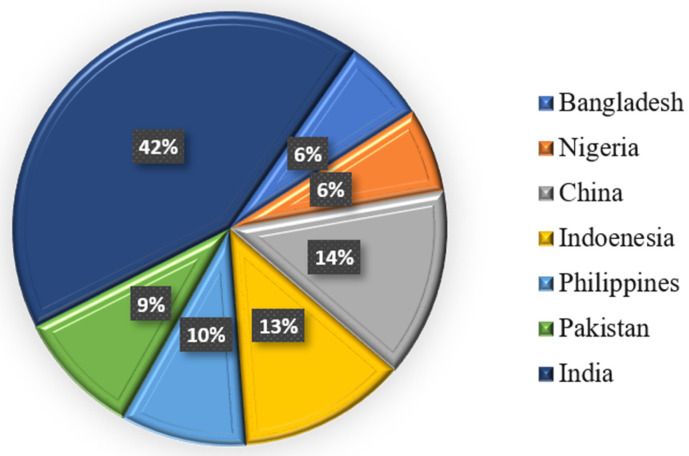
Percentage of diagnosed TB cases in seven different countries.

People with chronic TB are at high risk in their homes, workplaces, and social circles. The course of treatment requires the long-term administration of antibiotics [7]. Antibiotic resistance is becoming a greater problem as the occurrence of multiple drug-resistant tuberculosis (MDR-TB) increases. According to estimates from 2018, 25% of people globally may be latently suffering from TB. Approximately 1% of people experience an acute outbreak yearly [8]. After COVID-19, TB was the second most prevalent infectious disease-related cause of death in 2022, with an estimated 10.6 million active TB cases and 1.3 million deaths. As of 2018, seven nations accounted for more than half of all TB cases diagnosed (Figure 1) [9]. The bulk of TB cases were found in the WHO regions of South-East Asia (44%), Africa (24%), and the Western Pacific (18%). By 2021, the number of new cases per year decreased by roughly 2%. Approximately 80 percent of people in certain Asian and African countries test positive for TB, but just 5 to 10 percent of Americans test positive. Evidence from paleomicrobiological analyses of ancient human remains and genomic studies of Mycobacterium tuberculosis complex strains indicates that TB has impacted people for millennia [10].

### 1.2. Fundamental AI Methodologies Highlighted in This Review

This section succinctly elucidates some core AI approaches frequently referenced in tuberculosis control, explaining the specific AI technologies discussed in this evaluation. Recognizing the various applications in enhancing tuberculosis diagnosis, treatment, and drug discovery necessitates an understanding of methodologies such as machine learning (ML), deep learning (DL), artificial neural networks (ANNs), convolutional neural networks (CNNs), support vector machines (SVMs), and random forests (RF) [11].

#### 1.2.1. Artificial Intelligence

Artificial intelligence is a vast domain of computer science focused on developing machines or software that can perform tasks often necessitating human intellect [12]. This encompasses learning, problem-solving, pattern recognition, decision-making, and language understanding abilities. Artificial intelligence encompasses various techniques and applications, from rule-based systems to intricate learning models.

#### 1.2.2. Machine Learning

Machine learning (ML), an element of artificial intelligence, enables computer systems to autonomously identify patterns and make decisions or predictions based on data, without the need of manually programmed rules [13]. Machine learning techniques identify patterns in data to make predictions. They encompass a wide array of methodologies, including reinforcement learning (learning through rewards and penalties), unsupervised learning (detecting patterns in unlabeled data), and supervised learning (learning from labeled data). Machine learning approaches can be classified broadly.

- Support vector machines (SVMs) are supervised learning algorithms for classification and regression applications [14]. The fundamental objective of an SVM is to determine an optimal hyperplane—a decision boundary—that most effectively separates data points of various classes in a high-dimensional space. Diverse kernel functions enhance the flexibility and efficiency of SVMs in high-dimensional settings [15].
- Random forests (RF) are supervised ensemble learning algorithms that operate by constructing several decision trees during training and generate the mode of the classes for classification or the mean prediction for regression [16]. Bootstrapped aggregation (bagging) is the technique in which each tree in the forest is generated utilizing a randomly selected portion of the training data and a random selection of features [17]. This randomization facilitates generalization and mitigates overfitting. Due to its robustness, ability to handle high-dimensional data, and effectiveness in identifying critical predictive factors such as biomarkers and risk variables in TB diagnosis and treatment outcome prediction, random forests are extensively utilized in biomedical research.

#### 1.2.3. Deep Learning

Deep learning, or DL, is a subset of machine learning that employs artificial neural networks (ANNs) with several layers, termed “deep.” Deep architectures facilitate the model’s acquisition of hierarchical data representations, rendering deep learning very effective for complex applications such as image recognition, natural language processing, and pattern detection in large datasets [18].

- Artificial neural networks (ANNs) are computational models inspired by the architecture and function of the human brain [19]. Using weighted connections and activation mechanisms, they consist of interconnected layers of nodes, or “neurons,” that evaluate input data and recognize patterns. Deep learning is fundamentally based on ANNs, which are widely utilized in many biological applications, including pattern identification and disease classification [20].
- Convolutional neural networks (CNNs) are a specialized category of ANNs particularly designed for processing image input [21]. CNNs have demonstrated significant efficacy in autonomously extracting hierarchical features from imaging data, thereby facilitating the interpretation of chest radiographs in TB diagnosis [22]. The design, consisting of convolutional layers, pooling layers, and fully connected layers, facilitates the identification of complex spatial patterns associated with tuberculosis-related abnormalities such as cavities, nodules, and infiltrates. In high-burden, resource-constrained settings, CNN-based models trained on extensive datasets of annotated chest X-rays can achieve diagnostic accuracy that is comparable to or surpasses that of experienced radiologists, thereby facilitating rapid and affordable screening.

### 1.3. Significance of AI in TB Management

In high-burden regions, prompt and precise case identification remains a significant challenge in worldwide TB management. Consequently, enhancing the accessibility and efficacy of diagnostic tools, such as chest radiography (CXR), is gaining increasing significance [23]. Artificial intelligence (AI) offers a transformative answer for these issues. Artificial intelligence (AI) is a broad domain focused on developing intelligent systems. Machine learning (ML) enables artificial intelligence (AI) systems to learn from data, whereas deep learning (DL) is a specialized subset of ML that employs complex neural networks. AI-driven computer algorithms, particularly those employing deep learning (DL) and machine learning (ML) methodologies as detailed in Section 1.1, facilitate the rapid and accurate interpretation of chest radiographs, significantly aiding medical companies. This support may encompass improving the competencies of seasoned radiologists in demanding circumstances and facilitating initial screening interpretations through task-shifting in resource-constrained environments where specialists may be unavailable.

To achieve optimal accuracy and efficiency in TB diagnosis, these methodologies—ranging from random forests for predictive modeling utilizing patient data to convolutional neural networks for analyzing medical images like chest X-rays—are essential for identifying novel biomarkers and interpreting medical imagery. Research has shown that these artificial intelligence systems can identify TB from chest X-rays with sensitivity comparable to that of specialist radiologists, thereby assisting in prioritizing patients who require further confirmation tests [24]. Table 1 further illustrates the comparative advantages of AI-based diagnostic procedures over traditional methodologies, which are occasionally more time-consuming and less sensitive.

The significance of artificial intelligence in TB management extends beyond the first diagnosis. AI models are being developed to support critical activities such as monitoring side effects and predicting treatment results. Research is ongoing to utilize artificial intelligence and machine learning models to predict the likelihood of adverse outcomes such as mortality, treatment failure, or the onset of drug-induced side effects, including hepatitis, in the first stages of anti-TB treatment [25,26]. Such predictive capabilities offer more proactive and tailored patient care regimens. Furthermore, by enhancing the precision of image analysis and facilitating the detection of subtle disease signs that may be overlooked, artificial intelligence systems might serve as valuable adjuncts for radiologists and other healthcare professionals [27,28].

Despite these promising advancements, integrating artificial intelligence into standard TB therapy remains in progress. These technologies must be rigorously evaluated. Before the widespread use of computer-aided detection (CAD) systems in PTB diagnostic and screening programs, the World Health Organization (WHO) emphasized the need for more comprehensive data about their efficacy, equity, and practical implications.

**Table 1:** Comparison of traditional and advanced AI-based diagnostic methods.

Feature	Traditional Methods	AI-Based Diagnosis	References
**Accuracy**	50–70%	80–95%	[29]
**Sensitivity**	40–80%	85–98%	[30]
**Specificity**	60–85%	90–99%	[31]
**Turnaround Time**	days to weeks	seconds to minutes	[32]
**Cost**	High	Lower	[33]
**Human Dependency**	High	Low	[34]
**Scalability**	Limited	Highly Scalable	[35]
**Interpretability**	Expert-dependent	Data-driven	[13]
**Resistance Detection**	Requires molecular/genetic testing	Based on imaging and data, AI can detect resistance	[36]
**Application in Remote Areas**	Difficult	Feasible (AI-based mobile applications)	[37]

## 2. Traditional Diagnostic Methods for TB

### 2.1. Sputum Smear Microscopy

Sputum smear microscopy (SSM) is a diagnostic method of TB in underdeveloped nations, where over 95% of TB diagnoses and 98% of TB-related fatalities occur [38]. It is an easy, quick, and affordable method that is especially effective in areas where TB is common. Additionally, it identifies the most contagious patients and can be used in a wide range of socioeconomically diverse populations [39]. SSM technique does have some serious performance limits, though. The sensitivity is significantly reduced when there are fewer than 10,000 organisms of bacteria per milliliter of sputum [40]. WHO advises TB programs to use mWRDs to identify TB [41]. There is less sensitivity to positive results when bacteria are scanty, for example, in extrapulmonary TB. With a more than 70% sensitivity, fluorescent microscopy is among the most precise techniques for SSM [42]. However, 20–40% of instances still result in misleading negative sputum smear results. Due to its limited sensitivity and invasiveness (only 42–63%), bronchoscopy biopsy is typically not advised as a routine screening procedure, even though it can be utilized for additional study [43]. According to research, artificial intelligence (AI) offers some benefits in pathological diagnosis that can lower the rates of misdiagnosis and false negatives caused by human error [44]. A previous study examining AI-assisted pathology had higher sensitivity than conventional bacteriology. But it cannot distinguish drug-resistant TB from drug-sensitive TB [45].

### 2.2. The Culture of Mycobacteria

TB is typically confirmed bacteriologically by culture using liquid media that can be purchased commercially [46]. However, the expense, the infrastructure needed (biosafety level 3 [BSL-3]), and the lengthy turnaround time (around 3 to 6 weeks) are factors to be considered. Further, it is not a diagnostic test in highly infected countries [47]. However, traditional microscopy and culture are still required to track a patient’s reaction to therapy [48]. For an accurate characterization of non-tuberculous mycobacteria (NTM), culture is essential for detecting pediatric and extrapulmonary TB from paucibacillary samples [49]. Microscopy of the bacteria isolated from a patient sample, usually sputum, is a confirmatory test for TB. Mycobacterial culture is typically conducted in a liquid medium with indicators that enable the detection of bacterial growth through colorimetric or fluorometric methods. Automated techniques such as the BACTEC MGIT 960 and the Resazurin Microtiter Assay (REMA) assess the metabolic activity of Mycobacterium TB in real time by fluorescence detection, offering a more rapid turnaround compared to traditional solid medium cultures [50,51,52]. However, this process requires specialized equipment and facilities [53].

### 2.3. Nucleic Acid Amplification Tests (NAATs)

**Figure 2:**
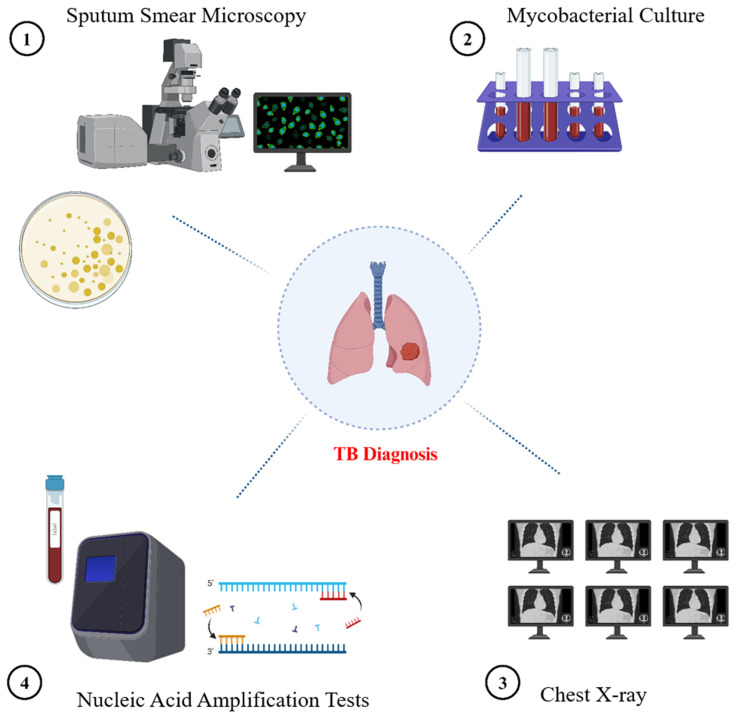
Overview of traditional diagnostic methods for tuberculosis. The image illustrates many approved methodologies for TB diagnosis: direct visualization of acid-fast bacilli via sputum smear microscopy; mycobacterial culture utilizing a liquid culture system (e.g., BACTEC MGIT tubes) typically employed for isolating Mycobacterium tuberculosis; chest X-ray (CXR) for detecting pulmonary abnormalities indicative of tuberculosis; and nucleic acid amplification tests (NAATs) for the molecular identification of Mycobacterium tuberculosis.

The use of NAATs has significantly improved TB diagnosis [54]. They have been on the market in the US for more than 20 years, and they are faster than culture and more accurate than sputum smear microscopy [55]. The primary goal of the direct amplification test, known as NAAT, is to enhance Mycobacterium tuberculosis-specific nucleic acid regions. Nearly all biological specimens can be used for these tests. Commercial kits of NAATs are accessible for household use [56]. According to recent research, NAAT has a good specificity and accuracy rate for pulmonary tuberculosis, which supports its capacity to rule out the illness. Additionally, its positive predictive value is high. Particularly in patients with positive sputum smear results, a high pretest likelihood is reasonably confirmatory of tuberculosis. Because of its great sensitivity, NAAT can be a useful method for verifying that M. tuberculosis is the cause of a positive sputum smear. However, patients with extrapulmonary tuberculosis and smear-negative pulmonary tuberculosis exhibit poor sensitivity [57]. Therefore, in these situations, a negative test does not rule out the diagnosis of tuberculosis and calls for further investigation. Additionally, NAATs cannot differentiate between viable and non-viable bacilli, and they are not useful tools for tracking how well a patient responds to treatment. Some traditional techniques are shown in Figure 2.

## 3. AI-Based Methods in TB Management: From Detection to Prognosis

AI models are becoming significant in several aspects of TB control. While AI significantly enhances the speed, reliability, and accessibility of TB diagnosis, its use also extends to predicting treatment outcomes. Research presented in Table 2 illustrates models developed for this purpose, indicating that AI may be utilized to forecast treatment efficacy. Another study by Kim et al. [58] evaluated the extent of tuberculosis from chest X-rays utilizing an AI-driven radiographic analysis, specifically employing an AI-based prognostic model known as DeepCatch X TB v1.0 to forecast treatment efficacy and culture conversion rates. Furthermore, artificial intelligence-driven digital adherence technologies (DATs) help ensure patients stick to their treatment regimens, which is crucial for preventing medication resistance [59,60]. AI facilitates the development of customized treatment programs by identifying patients at high risk of treatment failure or adverse drug reactions, potentially reducing the incidence of drug-resistant TB [61,62]. Some of the AI methods are explained below:

### 3.1. Chest Radiography (CXR)

Although no published evidence supports this, it is anecdotally believed that the chest radiograph, usually called the chest X-ray or CXR, is the most commonly conducted radiological investigation worldwide. The chest radiograph continues to be the imaging test that general practitioners most frequently seek, according to UK government statistics from the NHS in England and Wales [66]. Achieving the goals outlined in WHO’s End TB Strategy requires chest radiography, a crucial technique for the early detection of TB. The WHO has published a comprehensive overview of its guidelines for programmatic methods and recommendations for using chest radiography in TB detection [67]. When pulmonary TB cannot be verified bacteriologically, chest radiography, often known as a chest X-ray (CXR), is a valuable diagnostic tool for triaging and screening for the disease. Even while bacteriology has gained particular attention in contemporary diagnostic techniques, CXR can be utilized to choose people for referral for bacteriological analysis, and radiography continues to play a significant role in situations where bacteriological testing is unable to yield a definitive response [68]. In many places, access to high-quality radiography is restricted. When utilized as part of algorithms within the World Health Organization’s (WHO) recommended laboratory-based diagnostic tests, ensuring the broader and quality-assured use of CXR for TB detection can help with early TB diagnosis and possibly close the TB case-detection gap, a framework for strengthening the laboratory and health system [69]. Although significant, the traditional interpretation of chest radiographs (CXRs) for tuberculosis screening often faces challenges such as elevated radiologist workload, inter-reader variability, and restricted access to expert analysis in resource-constrained settings. AI-driven CXR research has emerged as a viable tool to address these limitations. Research, such as that conducted by Qin et al. [66], has examined the application of several AI techniques, including deep convolutional neural networks (CNNs), for the automated detection of tuberculosis using chest X-rays (CXRs). Typically including tens to hundreds of thousands of images, these artificial intelligence systems are trained on extensive datasets of annotated chest X-rays to comprehend radiological patterns indicative of tuberculosis. Subsequently, the AI analyzes new CXRs and produces an anomaly score or categorization, such as high or low risk of tuberculosis. In high-volume screening contexts, a significant advantage demonstrated by research is the ability of well-validated artificial intelligence algorithms to achieve diagnostic performance (sensitivity and specificity) comparable to or, in certain specialized tasks, surpassing that of human readers. This facilitates rapid patient triage and the identification of individuals requiring urgent microbiological test confirmation, thus reducing diagnostic delays and patient loss to follow-up. Furthermore, these tools facilitate task-shifting, enabling trained non-specialist healthcare personnel to do initial CXR screenings with AI assistance, aiding areas without radiologists. Despite the significant potential benefits regarding speed and accessibility, critical considerations encompass the disparity in performance among different AI tools, the imperative for robust local validation and calibration before implementation, and ongoing research to ensure demonstrable cost-effectiveness across diverse programmatic contexts [67,70].

It is essential to elucidate the dual role of artificial intelligence in interpreting chest X-rays for TB. AI is an auxiliary instrument for radiologists and physicians across diverse settings that emphasizes subtle findings, expedites workflow, and enhances diagnostic precision. AI-driven CAD systems are being evaluated for their potential to shift or replace tasks during initial screening and triage, particularly in remote or resource-constrained areas with insufficient expert readers [46,47]. In this context, artificial intelligence (AI) categorizes chest X-rays (CXRs) as probable or unlikely for tuberculosis (TB) to select patients for confirmatory testing, such as GeneXpert, facilitating screening programs in the absence of quick expert interpretation. Comprehending its utilization in diverse healthcare settings, especially remote locations, hinges on the distinction between artificial intelligence as an auxiliary aid vs. a primary interpretative instrument for triage [48].

### 3.2. AI-Assisted Diagnosis of CT Imaging

When detecting parenchymal lesions and displaying active characteristics of TB, such as cavities, parenchymal abnormalities, and lobular central nodules, CT is better than CXR [71]. However, compared to CXR, the enhanced diagnostic precision of CT imaging typically entails greater costs and reduced accessibility, particularly in resource-constrained settings. Regardless of its clinical advantages, these factors must be considered while evaluating its efficacy as a prevalent tuberculosis diagnostic instrument. While CT imaging offers superior information to CXR for detecting active TB characteristics and small parenchymal lesions, its interpretation can be complex and labor-intensive. The use of artificial intelligence in CT scans in tuberculosis diagnosis is driven by the need to leverage computational power for enhanced consistency, speed, and perhaps more profound analysis of these volumetric datasets [72]. Research, like that conducted by Yan et al. [73] has examined the use of 3D convolutional neural networks (3D-CNNs). These 3D-CNNs are particularly suited for CT data since they can analyze the whole 3D volume of the scan, allowing them to discern spatial correlations and features across several slices that may be overlooked by 2D analysis or human evaluation alone. In this research, AI models are trained to identify certain tuberculosis-related patterns, quantify lesion characteristics (e.g., size, volume, density), and differentiate tuberculosis from other pulmonary illnesses. These artificial intelligence systems provide potentially enhanced diagnostic accuracy and sensitivity, as well as the ability to offer objective, quantitative assessments of disease severity. Standardizing reporting, monitoring therapeutic response, and baseline assessment relies on this quantitative data and might prove advantageous. Challenges in artificial intelligence for CT include the necessity for extensive, well-annotated 3D datasets for training, the required computational resources, and the imperative to ensure that the AI’s findings are clinically pertinent and seamlessly integrated into radiologists’ workflows to enhance rather than supplant their expertise [72,74].

### 3.3. Molecular Diagnostics

Mortality and premature death can be decreased with a fast and correct TB diagnosis [61]. The objective of eradicating tuberculosis is threatened by the emergence of drug-resistant cases [75]. Significant progress has been made in laboratory diagnostic techniques for identifying infections. These tactics either supplement or take the place of the conventional methods that are currently in use [75,76]. Traditional techniques include microbial culture, acid-fast bacillus smear microscopy, while more sophisticated approaches include CRISPR–Cas, Gene Xpert, and LAMP. Despite being slow and labor-intensive, conventional diagnostic methods are widely employed in countries with elevated TB rates, mostly due to limited access to more advanced and expensive technologies [77]. Low sensitivity, low specificity, and limited access to the peripheral healthcare system where patients seek treatment are the main causes of the dearth of sophisticated procedures for identifying Mycobacterial infections. AI improves GeneXpert and TruNat molecular diagnostics by exploring deeper gene expression levels [78]. Machine learning tools enhance the efficiency of identifying resistance markers. Using high-throughput sequencing, learning tools improve the speed and reliability of detecting resistance markers in high-throughput sequencing data [79].

#### 3.3.1. CRISPR–Cas-Based TB Treatment

Recent advancements in CRISPR–Cas technology provide novel opportunities for rapid and accurate detection of infectious diseases such as TB [80]. CRISPR–Cas13a systems, recognized for their collateral cleavage activity, have been adapted into biosensing platforms capable of very sensitive and specific detection of nucleic acid sequences specific to Mycobacterium tuberculosis [81]. These devices employ RNA-guided detection systems to generate fluorescent or colorimetric readouts upon target recognition, thereby facilitating point-of-care diagnostics. Examining high-throughput data derived from fluorescence intensity, time-resolved cleavage patterns, and image-based outputs from microfluidic assays improved by ML techniques in augmenting CRISPR-based TB diagnoses. ML models typically built on SVM, RF, or CNNs may be trained to classify sample results, optimize threshold parameters, and differentiate between true and false positives based on signal features and kinetics. CRISPR–Cas systems are being examined for drug susceptibility testing by precisely identifying resistance-associated mutations in TB genomes, using ML algorithms to enhance variant interpretation through the integration of genomic, phenotypic, and clinical data [82]. Despite their significant value, the infrastructure demands and the need for extensive local validation continue to limit their potential application. In resource-constrained settings, integrating CRISPR-based diagnostics with AI and ML presents a powerful approach for the quick, accessible, and precise detection of TB [83].

#### 3.3.2. GeneXpert-Based TB Treatment

The WHO continues to endorse GeneXpert as one of active TB’s most common diagnostic techniques. It is the most sensible and dependable option due to its high specificity and incorporation into international TB initiatives [84]. By evaluating chest X-rays, detecting possible TB cases, and maybe forecasting treatment results, artificial intelligence (AI) can and is being used in combination with GeneXpert testing to enhance TB diagnosis and management [66]. By determining which people are most likely to benefit from testing, artificial intelligence (AI) can assist in maximizing the usage of GeneXpert, possibly lowering total costs and increasing efficiency [67]. Through workflow optimization, drug resistance prediction, and improved diagnosis accuracy, GeneXpert improves TB treatment. It reduces errors and ensures prompt detection of TB, including drug-resistant forms, by automating the interpretation of results. While real-time data processing links GeneXpert results with electronic medical records for quicker clinical action, AI-driven predictive algorithms evaluate patient data to determine severity and direct therapy choices [85]. AI also streamlines laboratory procedures, allowing for remote diagnosis and faster turnaround times. Examining medication resistance patterns also supports worldwide TB surveillance, eventually improving patient outcomes and effectively stopping TB spread [86].

#### 3.3.3. TB-LAMP

Based on the principle of loop-mediated isothermal amplification (LAMP), a TB detection kit was designed by Eiken Chemical Japanese Company for the TB detection [87]. This assay needs less than two hours, and the results can be visualized with the naked eye under ultraviolet light. It is considered a rapid diagnostic test kit for limited-resource lab settings because of its ease of use and limited infrastructure requirements [88]. By using AI, we can improve the accuracy, efficiency, and scalability of TB diagnosis. By integrating AI with the TB-LAMP approach, we can reduce the chances of error in the results by using automated fluorescence detection through image recognition. AI helps medical professionals with result evaluation. Further, the reaction conditions of LAMP can be optimized through machine learning algorithms to increase the sensitivity and specificity [89]. TB-LAMP data can be evaluated and retained through the AI-powered applications for extensive epidemiological research and effective disease control.

### 3.4. Smartphone-Based Imaging Devices (SIDs)

Numerous biomedical applications have demonstrated the versatility of smartphone-based imaging devices (SIDs) [90]. Technological interventions, including portable gadgets, can be employed in remote settings for high-quality medical services [91]. Optical microscopy is a common method for testing sputum samples for TB; other significant processes include PCR and culture assays [92]. In contrast to the PCR-based approach, which necessitates costly equipment and high-end resource settings, culture tests are difficult to execute and require a prolonged time in incubation [93]. Thus, the most popular method for diagnosing TB is SSM. Therefore, developing screening techniques based on microscopes could greatly aid in diagnosing tuberculosis. High sensitivity has been demonstrated using fluorescence microscopy compared to bright-field microscopy [90]. As light sources for fluorescence microscopy have improved, the latter is becoming more affordable and frequently used. Chang et al. created Cell Scope, a fluorescence microscopic system for smartphone TB detection [94]. The device comprises 3D-printed components and an inkjet-printed lens connected to the phone’s camera [95]. The samples were excited using several LEDs with fluorescent filters. The sample stage’s immobility made it impossible to use the lens to change the sample’s focus [96]. Support vector machines (SVM) can distinguish between positive and negative TB items with an average precision of 89.2%  ±  2.1%, making it possible to diagnose TB from sputum smear samples more quickly and accurately [39].

### 3.5. Nano-Technological Multimodal Diagnosis of TB

**Figure 3:**
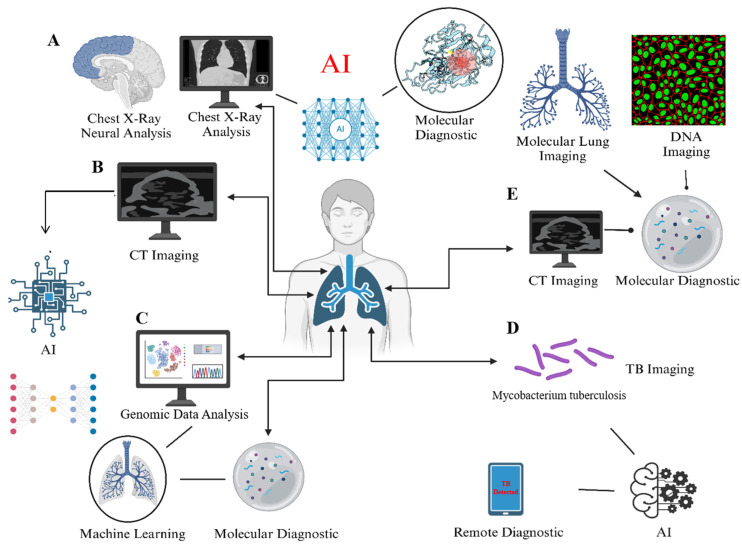
Conceptual framework illustrating critical data areas in TB management where AI can be applied. This illustration highlights potential integration points for AI and ML, highlighting the many data modalities involved in TB diagnosis and treatment, including (**A**) chest X-ray imaging, (**B**) CT scans, (**C**) genetic data, (**D**) bacteriological evidence, and (**E**) molecular diagnostics. The illustration is intended to provide a broad visual representation of the kind of data generated throughout TB care pathways where AI can be beneficial, rather than presenting analytical results.

Utilizing the unique properties of nanoparticles (NPs), nanomedicine is breaking new ground in contemporary healthcare by rethinking diagnostics and treatment [97]. Undoubtedly, using nanoparticles (NPs) in this treatment is promising and provides remarkable results for overcoming the illness. Different nanocarriers were created for medication delivery applications using various administration techniques [98]. One advantage of using a drug-loaded NP in TB treatment over traditional medication therapy may be the ability to control and maintain the drug release. Additionally, the drug-encapsulated NP can reduce dosing requirements and address issues related to inadequate compliance [99]. While the treatment system has grown, NPs have been used in diagnostic and therapeutic procedures over the last ten years. Nuclear imaging, optical imaging, ultrasound, magnetic resonance imaging, and computed tomography, which encompasses single-photon computed tomography and positron emission tomography, were among the applications for which these “theragnostic” NPs were created [100]. As a result, it is also essential to identify latent TB and stop its reactivation [101]. Some of the AI-based methods are illustrated in Figure 3.

## 4. AI in Treatment Monitoring

Artificial intelligence (AI) applications that use sophisticated deep learning techniques for picture identification jobs can automate medication adherence monitoring and improve efficiency [33]. AI has not received much attention regarding clinical medication adherence monitoring. Even in places with low resources, like Africa, AI has the potential to revolutionize the delivery of healthcare [25]. Table 3 summarizes the implementations of AI in different countries and their results.

### 4.1. Adherence Tracking

In several healthcare settings, digital adherence technologies have quickly become instruments for enhancing care delivery [105]. WHO released guidelines on video-based directly observed therapy (VDOT) in 2017 and approved it as a viable substitute for directly observed therapy in tuberculosis treatment [107]. VDOT eliminates geographical barriers because it allows medical professionals to remotely monitor patients’ medication intake patterns, particularly in populations that are difficult to access [108]. Because patients can decide where and when to take their TB medications, it also increases autonomy [109]. WHO has approved DATs, which have been used on a local and large scale in various contexts and have a growing body of data supporting person-centered care in TB [110]. According to researchers, the use of AI in the healthcare industry has the potential to revolutionize several clinical practice areas, including medical imaging. This technology has greatly improved the effectiveness of care delivery through properly organizing workflows within the healthcare system [111]. Therefore, to hasten broad acceptance and impact, digital adherence technologies can be combined with more sophisticated tools like artificial intelligence (AI) [112].

### 4.2. Monitoring Side-Effects of Treatment

Treatment for tuberculosis involves a multi-month course of medication. Treatment failure, treatment interruption, and poor adherence are all linked to adverse effects (AEs). To reduce resistance, early detection and treatment of AE are crucial. Alternative medications may need to be taken in certain situations, and the medicine and/or regimen may need to be completely stopped [33]. This offers crucial information about how AI technology might improve ATTB therapy management and monitoring [113]. From 2013 to 2023, a comprehensive search across six databases investigated the use of AI to predict the effectiveness of PTB treatment. Convolutional neural networks and SVM are excellent at predicting how long a therapy will last, while random forests, artificial neural networks, and classification and regression trees are promising for predicting negative reactions and results [114]. Drug resistance can be accurately predicted using RF and NN. Improvements in AI provide better patient outcomes, enhanced monitoring techniques, and open the door for more AI studies in PTB treatment monitoring [115]. RF, SVM, CNN, LR, and other models have become focal points of study with promising application potential when AI is applied to predict treatment duration, efficacy, and DR of ATTB therapy [116]. According to recent studies, the CNN model has the lowest MAE value among these models, which makes it the recommended option for assisting doctors in deciding on the specific treatment durations for PTB patients in upcoming clinical investigations [117].

## 5. AI in TB Drug Discovery

**Figure 4:**
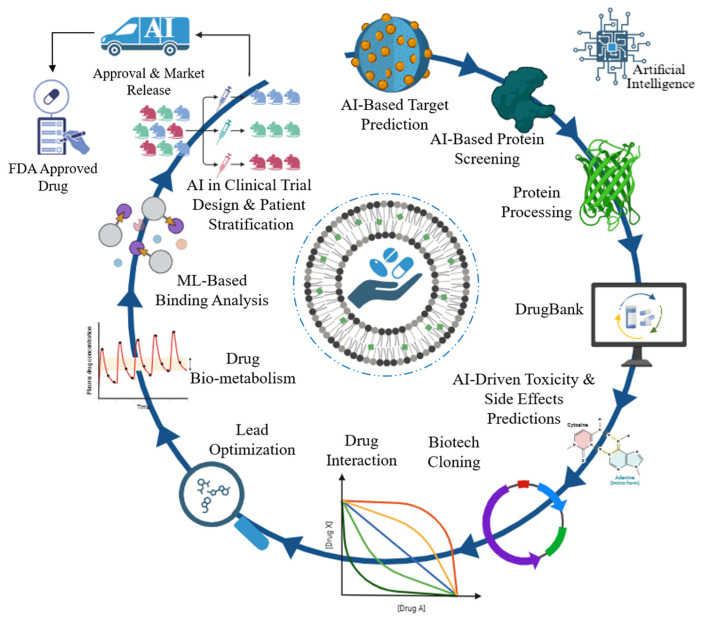
Artificial intelligence (AI) in the anti-tuberculosis drug development process at integration. This image illustrates the enhancement of traditional drug development methods with artificial intelligence and machine learning (ML). Significant phases augmented by artificial intelligence and machine learning encompass AI-based target prediction, AI-based protein screening, AI-driven toxicity and side effects predictions, ML-based binding analysis, and AI in clinical trial design and patient stratification. These computational techniques aim to accelerate the testing, identification, and optimization of novel anti-TB therapies.

Effective control of TB has several obstacles, and the existing worldwide targets for disease burden reductions appear unachievable [118]. Patients experience significant physiological and social side effects from lengthy treatment plans, and it can be challenging to obtain reliable, consistent diagnoses due to complex pathophysiology and technical limitations [119]. AI applications in healthcare have greatly enhanced basic research, diagnosis, and therapy [120]. The existing and possible uses of sophisticated AI models in global TB control and the ramifications of using these tools within the public health community are necessary [121]. Illustration diagram of AI in anti-TB drug discovery (Figure 4).

### 5.1. Identifying Novel Drug Targets

The control of TB faces a lot of challenges because of the growing prevalence of drug-resistant Mycobacterium tuberculosis (Mtb) [122]. To treat drug-resistant TB and decrease the duration of normal therapy, new anti-TB medications must be developed immediately. A theoretical basis for creating new drugs will be established by identifying pharmacological action targets [123]. Identifying novel targets and the corresponding inhibitors has advanced significantly with the growth of molecular biology and the accomplishment of Mtb genome sequencing [124]. Despite the potential advancements that AI-based analysis tools offer, opinions on AI in the medical industry remain a significant topic of dispute. Using natural language models, a subset of DL models, to automate administrative duties for nurses and clinicians led to 17% and 51% reductions in working hours, respectively [125]. Eighteen new anti-tubercular drugs are currently undergoing phase I–III trials. The complexity of the TB poses a major obstacle to the rate at which new compounds are identified and developed; the use of ML techniques for large-scale screening and compound prioritization procedures has the potential to expedite the TB drug development pipeline and offer a new tool in the fight against drug resistance [126]. Recent developments in fundamental studies of host–pathogen interactions provide fascinating illustrations of the new information that can be gleaned from complicated data-generating investments in tuberculosis research [127]. Novel molecular approaches, such as single-cell multi-omics, have enabled improved resolution of the underlying cellular dynamics of disease and response to treatment in various infectious diseases [122,124]. These techniques yield high-dimensional datasets rich in opportunities for host-directed therapy or new pharmacological target pathways [119]. However, powerful neural networks are needed to extract significant biological signals from these data.

### 5.2. Virtual Screening of Compounds

Finding active molecules with strong binding potential for particular disease-specific targets is common in drug discovery projects [128]. The ChEMBL database was used to organize the first antimycobacterial bioactivity dataset. After training six machine learning models for predicting anti-Mtb bioactivity, an ensemble approach was used to incorporate well-established machine learning methodologies. The high number of anti-TB compounds can be predicted accurately after compounds in the Drug Bank database were tested for anti-Mtb drug repurposing [126]. This AI-driven approach for the quicker, more economical development of innovative TB treatments would be validated by identifying first-in-class inhibitors of underutilized targets with strong bactericidal efficacy against Mtb [129]. The course of the disease in the infected person and the population-level transmission of Mtb can be tracked using a combination of these techniques. To determine treatment choices, it is crucial to identify drug-susceptible or resistant variants of Mtb as soon as possible. Traditionally, this has been performed through culture-based susceptibility screening, which is costly and time-consuming [124]. The complete genome sequencing of this intricate microbe will reveal not just medication susceptibility but also the genetic islands of resistance and susceptibility [119]. Given the opportunities, resources must be allocated to developing a standardized bioinformatics pipeline for diagnosis and establishing a secure and understandable environment for high-end computation, data processing, and sharing. Attempts have already been made in this direction. A machine learning approach called Extreme Gradient Boosting (XGBoost) improves the model’s prediction ability by using a stage-like arrangement of decision trees [130]. Draggable target proteins in humans have already been identified and predicted using this approach. Protein sequences were used as input for the model, grouped by dipeptide composition and pseudo amino acid composition segmentation [131]. A thorough use of the methodology for identifying potential targets for anti-TB medication at various infection stages is still pending.

### 5.3. Drug Repurposing

Computer-aided drug designing is a major contribution to discovering drugs for neglected tropical diseases using machine learning, improving the precision of these techniques, and reducing obstacles to entry for investigators [132]. Because Mycobacterium TB strains (M. tuberculosis) are becoming drug-resistant so quickly, it is difficult to develop potential anti-tubercular compounds [133]. Researchers can find hidden connections between existing drugs, disease targets, and potential remedies by utilizing AI to analyze massive databases. For more than ten years, computational machine learning techniques have been extensively used to search for antibacterial drugs. The GSK antimalarial dataset’s compounds were scored (repurposed) using these AI-based tools based on whole-cell screening data from Mycobacterium tuberculosis [134]. Bayesian machine learning models performed similarly to deep neural networks using external test sets. Although real high-throughput screenings were conducted more than ten years ago, hundreds of new non-antibacterial medications have since been approved; however, many of them have likely never been tested against Mycobacterium tuberculosis, which presents a potential future repurposing opportunity. These AI-based techniques could virtually screen FDA-approved medications [135]. However, AI’s demonstration of the addition of bedaquiline or delamanid is a promising trend toward lowering mortality risk in the early stages of MDR-TB treatment [136]. One effective result of employing virtual screening to find an anti-TB medication that inhibits the ATP synthase enzyme is bedaquiline [137]. There are now more chances to find new chemical scaffolds that target particular proteins thanks to the development of computational methods [138].

## 6. Challenges and Limitations

There will be a significant and disastrous resurgence of tuberculosis due to the growing resistance strains. Anti-tubercular drug resistance is lessened by recent developments in TB treatment [24]. Because Mycobacterium tuberculosis is so adaptable, drug resistance in tuberculosis is a complicated phenomenon. These bacteria may become resistant to anti-TB medications through efflux pumps, genetic changes, and other processes. As a result, conventional TB treatment plans, which frequently include a mix of drugs, might no longer work against bacteria that are resistant to drugs [139]. Effective TB disease control has several obstacles, and the existing disease targets for burden reduction appear unachievable. Long treatment regimens have major physiological and social repercussions for patients, and the combination of complicated pathophysiology and technological constraints makes it difficult to obtain consistent, trustworthy diagnoses [140]. AI in healthcare has greatly advanced basic research, diagnosis, and therapy. However, infrastructures prioritizing thorough data gathering and collaborative research environments that encourage stakeholder engagement are essential to their success [24,139]. These include limited data quality and availability, a lack of standardization, bias, and ethical considerations. Furthermore, the features of AI-assisted diagnosis, such as its incapacity to thoroughly examine patients’ clinical symptoms, focus on identifying minor lesions, and minimize missed diagnoses, may result in comparatively high false-positive outcomes [71]. The issues above must be resolved to optimize the potential of AI-assisted diagnosis in enhancing precision, effectiveness, and patient outcomes. Doctors, researchers, legislators, and regulatory agencies must work together to overcome these obstacles and ensure the effective incorporation of AI into standard clinical practice for PTB diagnosis [141].

Some studies indicate that the use of AI and machine learning to forecast the length of TB therapy is restricted, requiring enhancements to the popularity and robustness of current models [140,142]. Models that predict treatment results and adverse reactions with above 80% accuracy include RF, XGBoost, LR, ANN, RP, Bagging, and SDLM. SVM shows great clinical promise while marginally less effective, with over 70% accuracy. The included variables and parameters affect the model’s accuracy [143]. For example, Rosenfeld G et al. report an accuracy range of 74% to 84%, whereas Liao KM et al. claim that the RF model achieves 85.6% accuracy. Low sensitivity (<50%) must be addressed despite excellent specificity. A major area of focus is MTB resistance prediction, where the accuracy of DCNN and CNN + SVM models exceeds 80%. With an AUC value above 0.9, the SVM, RF, ensemble of classifier chain (ECC), multi-label K-nearest neighbor (MLKNN), and deep denoising auto-encoder (DeepAMR) models demonstrate interesting applications. Based on the RF classifier, Li Y et al.’s nodule model and combined model (TIB and nodules) perform exceptionally well, with an accuracy (>80%) and AUC (>0.9) [144].

However, some of the challenges include the following: The caliber and variety of training data have a significant impact on AI algorithms [24,139]. For algorithms to work well, representative datasets that cover a range of demographics and illness presentations are essential, and overcoming organizational, legal, and technical obstacles is necessary to integrate AI solutions into current healthcare systems [141,143]. Widespread adoption depends on user acceptability and smooth integration, AI applications in healthcare bring up moral questions about algorithm openness, data security, and patient privacy. The inherent need for high-quality, large-scale, and diverse datasets is a barrier to the effective application of artificial intelligence in TB therapy. While effective AI models depend on large data volumes, the quality of this data—including aspects like exact annotations, standardized imaging techniques, thorough clinical information, and low noise—is of great relevance. Under low data quality, AI models may produce “garbage in, garbage out” situations whereby their therapeutic efficacy is reduced by erroneous, biased, or bad predictions. Furthermore, challenges in data availability remain marked by limited access to large-scale, representative datasets from several geographical locations and different patient groups. Data silos, marked by the dispersion of significant information among different organizations or systems without interoperability, impede the progress of generalizable artificial intelligence solutions able of consistent performance across many populations and healthcare situations. It is critical to address these issues in order to promote confidence and guarantee the responsible application of AI [71,142].

## 7. Future Directions

In the healthcare industry, where vast volumes of data are generated and the possibility of effectively sorting them to enhance patient outcomes is especially alluring, the enormous computational and storage capacity of digital technologies has significant ramifications [145]. Although to varied degrees, human experts are still needed to supervise many of the healthcare procedures that digital technologies have expedited. Traditional examples include human-moderated online discussion groups devoted to therapeutic issues like drug-resistant tuberculosis. Medical experts have not yet been replaced by robots, despite the fact that they have developed to execute some duties better than their teachers [61]. Because they rely so significantly on the interpretation of disease appearance, medical specializations like imaging, microscopy, and pathology are frequently requested in pulmonology and tuberculosis [146]. AI systems can analyze chest X-rays and CT scans to identify TB-related abnormalities, such as cavities and nodules, that could indicate MDR or XDR TB. AI makes it easier to understand complex patterns and detect drug resistance more rapidly and accurately than manual methods [147]. The traditional techniques for diagnosing tuberculosis frequently have drawbacks, including laborious processes, inconsistent accuracy, and reliance on trained staff. On the other hand, AI presents intriguing ways to boost early detection, increase precision, and optimize treatment plans, which could completely transform TB management globally [141]. A significant shift in healthcare is represented by the application of AI to tuberculosis diagnosis, which offers revolutionary advantages in terms of precision, effectiveness, and accessibility [148]. Enhancing the resilience of AI algorithms to improve diagnostic technologies, and tackling ethical and legal issues are some future research avenues [71]. Effective guidelines for converting results into practical suggestions while taking individual patient characteristics into account are necessary for integrating AI into healthcare decision-making. Future research should strive for thorough validation, ongoing enhancement of high-precision models, and practical recommendations for clinical practice [117]. The main ethical issues with AI in PTB treatment monitoring are privacy violations. Data pertaining to tuberculosis patients is sensitive; therefore, stringent data security and privacy regulations are necessary, presenting significant real-world challenges. It is imperative that patient information is secret and ethically managed during data collection, storage, processing, and dissemination. This involves implementing robust data governance structures where appropriate, utilizing sophisticated anonymization or de-identification techniques, and ensuring secure data transmission and storage infrastructure. A significant concern is navigating the intricate web of ethical and legal dilemmas. Compliance with national and international data protection regulations (such as GDPR, HIPAA, or local equivalents) regarding patient permission, data ownership, and cross-border data transfer must be meticulously observed. The ethical implications of artificial intelligence decision-making necessitate ongoing scrutiny and the development of transparent and accountable AI systems, particularly concerning biases derived from historical data that may exacerbate health disparities. Instilling confidence and ensuring patient safety relies on clearly established legislative pathways for the assessment and certification of AI technologies in TB treatment. Although AI forecasts effectiveness, concerns about data storage and informed permission arise. Algorithms’ limited accuracy raises concerns about potential harm and accountability. Patients’ and clinicians’ skepticism stems from their incomplete knowledge of AI in this area [149]. Prioritizing patient data privacy, confidentiality, equity, and transparency is necessary to address ethical issues. It is essential to set up a safe medical data system and governance structure. Strong, flexible models require ongoing improvement and validation in a variety of populations. Furthermore, attempts to introduce more advanced methods for detection and treatment are crucial to the eradication of tuberculosis [146,147]. These more recent techniques have the potential to significantly enhance management algorithms in high-burden facilities.

Future research and implementation recommendations must clearly delineate the specific role of artificial intelligence tools in various therapeutic pathways, whether as a primary reader for triage, a secondary reader for quality assurance, or an auxiliary tool integrated into expert workflows. Defining context-specific use cases, particularly in remote or resource-limited settings, is essential for ensuring ethical and effective implementation, which mitigates concerns around the replacement of critical human oversight with appropriate technological applications to enhance healthcare accessibility.

## 8. Conclusions

TB is an infectious disease with high mortality rates worldwide, particularly in underdeveloped nations. In immunocompetent people, TB is usually confined by granulomas, resulting in a latent TB infection. TB infection can reappear when host immunity is weakened, which can result in a high bacterial burden and the progression of the disease, which in turn causes clinical symptoms and the spread of TB. In developing nations with limited access to sophisticated radiological knowledge, AI may help with TB diagnosis and prognosis. This technology may also be helpful in tracking TB cases and forecasting the efficacy of treatment plans and acute side effects during therapy. In order to predict active TB and guarantee the effectiveness of treatment, genetic screening can also play a critical role. Despite the benefits indicated above, there are a number of possible knowledge gaps and areas that require further investigation in relation to TB care and the functions of AI, genetic screening, and WGS. In order to successfully incorporate AI into clinical practice for the management of tuberculosis, a comprehensive strategy that takes into account usability, technological, regulatory, and educational factors is needed. Research in these fields may open the door to a more extensive and successful application of AI to treat tuberculosis and enhance patient outcomes.

## Figures and Tables

**Table 2:** AI models forecasting the results of TB treatment.

Studies	AI Models	Important Results	References
An Analysis of Six Countries Using Machine Learning to Forecast Treatment Performance in Tuberculosis	Artificial Neural Networks, Random Forests, and Support Vector Machines	The random forests model predicted treatment results with 76% accuracy The support vector machine model’s specificity was 95.71%, and its accuracy was 73.05% The sensitivity of the artificial neural network was 68.5%	[16,63,64]
A Multicenter Cohort Study Using Artificial Intelligence-Based Radiographic Extent Evaluation to Forecast Tuberculosis Treatment Results	AI-Powered Radiography Evaluation	AI-derived evaluations of the degree of tuberculosis from chest X-rays significantly predicted treatment efficacy and culture conversion rates Poorer outcomes and more severe disease were associated with higher extent ratings.	[24,65]

**Table 3:** Summarization of AI applications in different countries.

Countries	AI Implementation	Influences	References
Vietnam	AI software combined with X-ray analysis of the chest	Enhanced chest X-ray interpretation quality, which resulted in the discovery of more TB patients during community screening initiatives	[102]
India	Wadhwani AI’s AI technologies for TB screening and forecasting outcomes	Improved TB screening procedures and forecasted patient results, which helped develop more potent treatment plans	[103]
Myanmar	AI-powered study of chest X-rays	Improved patient care by addressing the lack of radiologists and expediting TB diagnosis	[104]
Africa	AI-powered drug adherence monitoring system	Assessed patient compliance with TB therapy, helping to guarantee the efficacy and completion of treatment	[105]
United States	AI-powered radiography rating to forecast the effectiveness of treatment	Culture conversion and treatment outcome predictions for pulmonary tuberculosis patients, supporting individualized treatment planning	[106]
